# Magnetic interactions between metal sites in complex enzymes

**DOI:** 10.1007/s00775-025-02120-1

**Published:** 2025-07-24

**Authors:** Biplab K. Maiti, Isabel Moura, José J. G. Moura

**Affiliations:** 1 School of Sciences, Department of Chemistry, Cluster University of Jammu, Jammu, 180001 India; 2 LAQV, NOVA School of Sciences and Technology, Campus de Caparica, 2829-516 Caparica, Portugal

**Keywords:** Magnetic interactions, Electron transfer, Iron–sulfur centers, [NiFe] Hydrogenase, Aldehyde oxidoreductase, CO dehydrogenase

## Abstract

Magnetic interactions between iron–sulfur (Fe/S) clusters and transition metal centers such as nickel, molybdenum, and copper play a central role in the function of key metalloenzymes. These interactions, which arise from electronic coupling, spin exchange, and spatial arrangement, directly influence redox behavior and catalytic efficiency. This review highlights three distinct complex enzymes—[NiFe] hydrogenases, mononuclear molybdenum-containing xanthine oxidase (XO) family, and [NiFe] and [MoCu] carbon monoxide dehydrogenases (CODHs)—as paradigms for understanding (Fe/S)-metal center interactions. In [NiFe] hydrogenases, (Fe/S) clusters serve as electron relays that magnetically interact with the catalytic [NiFe] active site. In XO-type enzymes, a mononuclear Mo center is functionally and magnetically coupled to nearby Fe/S clusters, modulating substrate reduction and electron transfer. Similarly, in CODHs, both [NiFe]—and [MoCu]-dependent variants exhibit strong magnetic communication between metal active sites and surrounding Fe/S clusters, crucial for CO_2_/CO interconversion. Advanced spectroscopic approaches, particularly electron paramagnetic resonance (EPR) and related techniques, combined with theoretical modelling, have provided deep insights into the electronic structures and dynamic interactions within these metalloenzymes. Understanding these magnetic interactions not only sheds light on fundamental electron-transfer and enzymatic mechanisms but also guides the design of bioinspired catalysts and energy-conversion technologies.

**Graphical abstract:**

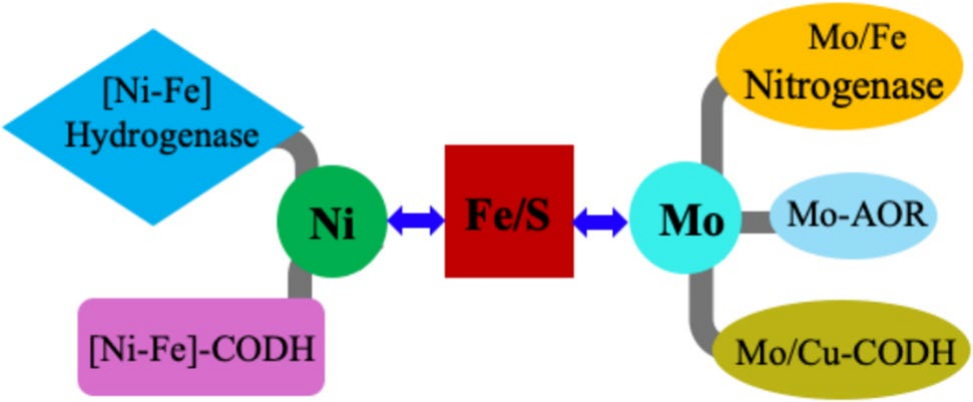

## Introduction

Magnetic interactions between iron–sulfur (Fe/S) clusters and other active metal sites, such as nickel, molybdenum and copper, are crucial for the catalytic activity and structural stability of many metalloenzymes including nitrogenases, hydrogenases, carbon monoxide dehydrogenase (CODH) and mononuclear molybdenum enzymes, which play essential roles in key biological processes such as nitrogen fixation, hydrogen metabolism, carbon processing, and sulfur assimilation [1–10].

The interplay between Fe/S centers and active-site metals is mediated by electronic coupling, spin exchange, and structural proximity, which influence the redox properties and reactivity of these enzymes [1–17]. This review focuses on bacterial [NiFe] hydrogenases (highlighting nickel’s interaction with Fe/S centers) [7, 13, 16], and molybdenum enzymes, ranging from mononuclear forms to more complex systems and examining molybdenum interactions with other metal centers: specific examples include the xanthine oxidase enzyme family (such as aldehyde oxidoreductase) [8, 17], and CODH ([MoCu] and also [NiFe] variants) [11, 12].

Advances in understanding these magnetic interactions are driven by spectroscopic techniques such as electron paramagnetic resonance (EPR) and related techniques, and computational modelling. Insights gained from these studies enhance our knowledge of metalloenzyme function, guiding the design of biomimetic catalysts and promoting applications in sustainable energy and biotechnology. These findings underscore the intricate role of magnetic interactions in fine-tuning enzymatic performance, bridging the gap between bioinorganic chemistry and functional enzyme engineering.

## [NiFe] Hydrogenase, a special class of hydrogen-metabolizing enzymes

**Fig. 1:**
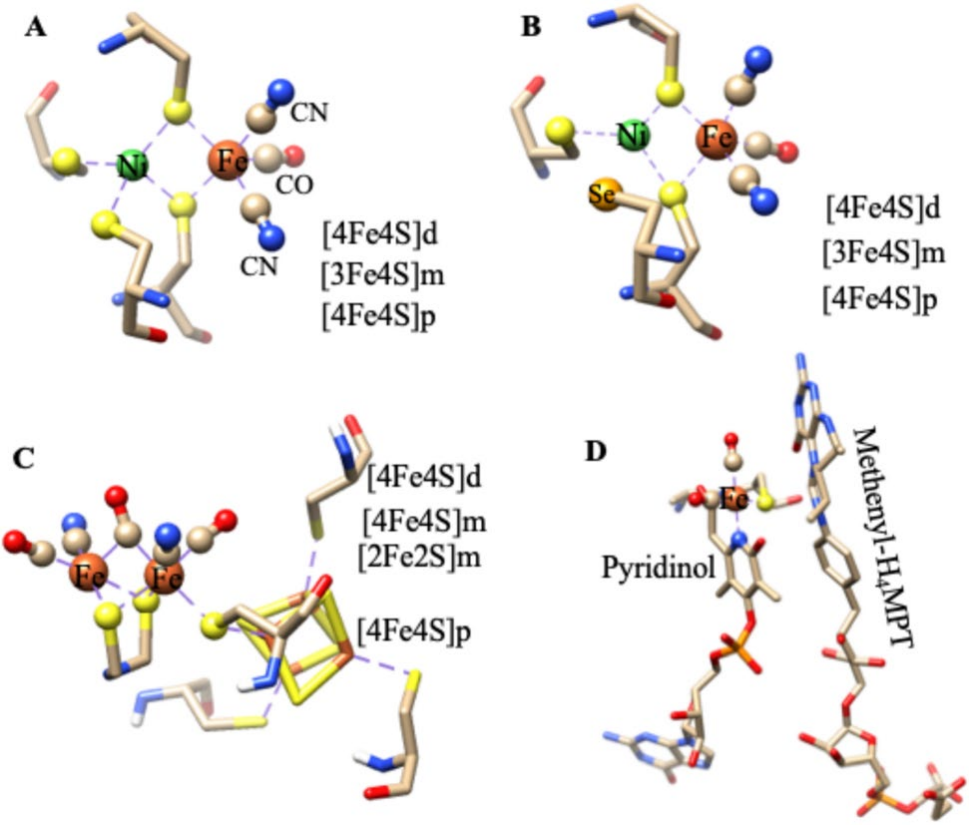
3D structures of active sites of three types of hydrogenases (Hases): **A** [NiFe]-Hase from *Desulfovibrio gigas* (PDB: 1YQ9), **B** [NiFeSe]-Hase from *Desulfomicrobium baculatum* (PDB:1CC1) **C** [FeFe]-Hase from *Clostridium pasteurianum* (PDB: 4XDC), and **D** Fe-Hase from *Methanococcus aeolicus* (PDB:6HAV). [4Fe4S]d: distal iron-sulfur, [3Fe4S]m: mediator iron-sulfur, and [4Fe4S]p: proximal iron-sulfur cluster

Nature possesses a class of enzymes, notably hydrogenases (Hases), which reversibly cleave molecular hydrogen into electrons and protons: H_2_ $\leftrightarrow$ 2e^−^  + 2H^+^ [7]. Hydrogenases are generally classified as iron-sulfur-containing proteins, with iron atoms arranged in different cluster configurations [7]. At least, three different types are now recognized within this bacterial group: [FeFe] hydrogenases, contains several [4Fe-4S] clusters and an active site considered an atypical Fe/S cluster termed the “H-cluster” [7, 18, 19]; [NiFe] hydrogenases with one nickel and iron-sulfur centers generally arranged as one [3Fe4S] and two [4Fe4S] clusters [7, 20, 21] (subgroup [NiFeSe] hydrogenases with iron-sulfur centers and equimolecular amounts of nickel and selenium (Ni bound to a selenium cysteine)) found in bacteria and archaea [7, 22, 23], and the third class of hydrogenases, [Fe]-hydrogenase contains a mononuclear iron center with no Fe/S cluster found only in some *hydrogenotrophic methanogenic* archaea [24]. The structures of metal active sites of various hydrogenases are depicted in Fig. 1. Among them, [NiFe] Hases are the main focus of this article.

**Fig. 2:**
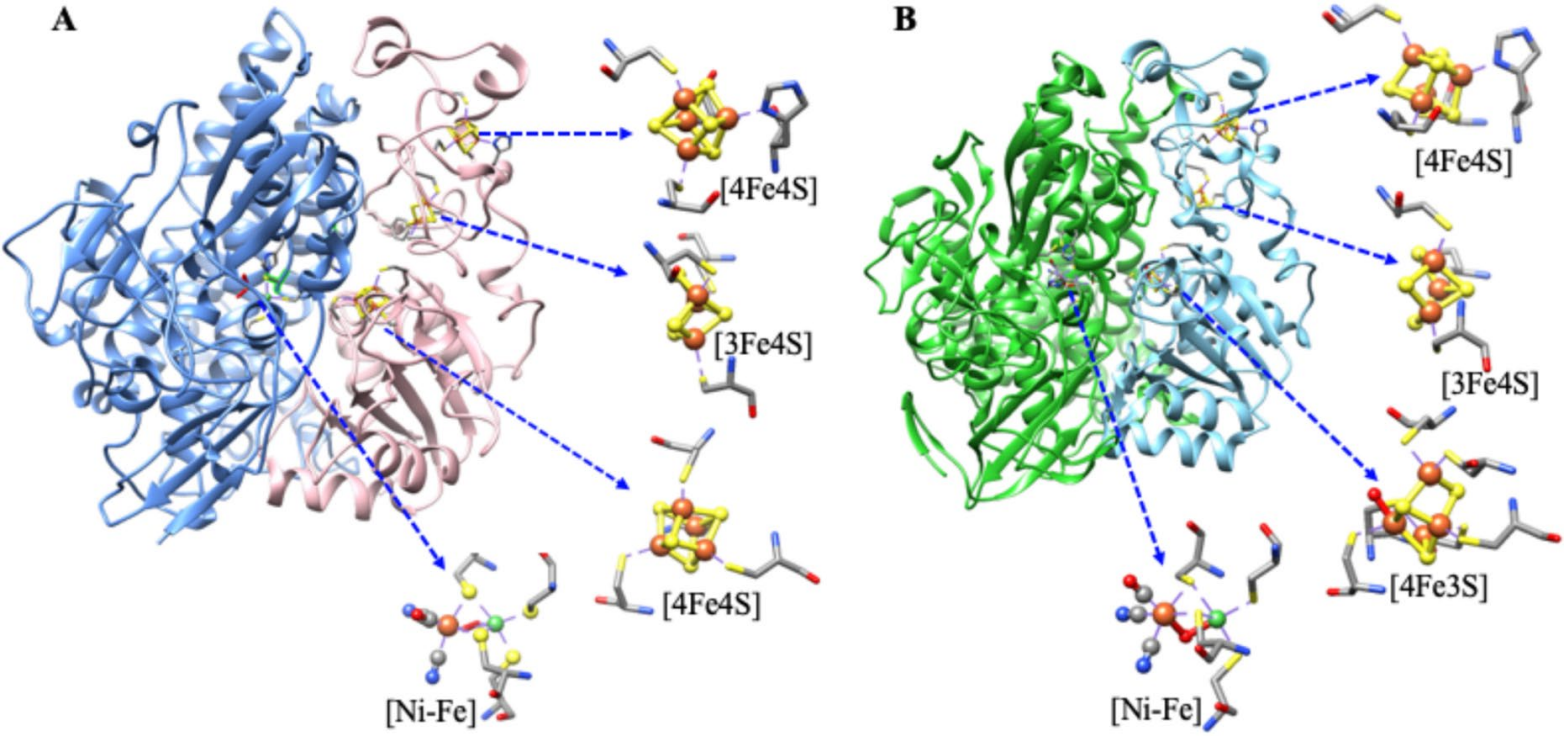
**A** O_2_- sensitive) [NiFe] hydrogenases from *Desulfovibrio gigas* (PDB: 1YQ9), and **B** O_2_-tolerant [NiFe] hydrogenases from *Ralstonia eutropha* (PDB: 4IUB). Highlighted the active site as well as iron-sulfur clusters

[NiFe] hydrogenases are subdivided into two subgroups: (a) O_2_-sensitive [NiFe] hydrogenases that are inhibited under oxic conditions, and (b) O_2_-tolerant [NiFe] hydrogenases that can sustain activity under oxic conditions [7, 21, 25, 26]. The 3D structural features of both O_2_-sensitive and O_2_-tolerant [NiFe] hydrogenases are shown in Fig. 2. Both have similar active site structures, but the main difference is found in the proximal iron-sulfur cluster, [4Fe3S]-6Cys cluster in O_2_-tolerant and classical [4Fe4S] cluster in O_2_-sensitive-hydrogenase. The binuclear active site, [NiFe], is attached to the protein chain by four cysteine residues. Two cysteines bridged the metal centers (Fe and Ni), and the other two cysteine residues were terminally coordinated to the nickel site. In addition, biologically unusual CO and CN^–^ ligands are coordinated to Fe-ion in active sites and make them fascinating examples of ‘organometallic’ cofactors [7, 21, 25, 26].

The [NiFe] Hydrogenases isolated from sulfate-reducing bacteria, particularly *Desulfovibrio* (*D.*) species, have served as model systems. In particular, extensive work has been performed on the hydrogenase isolated from *Desulfovibrio gigas,* a prototype of the [NiFe] hydrogenases [27–36], and the 3D structure was first reported, in 1995, by Volbeda and co-workers [21, 25]. *D. gigas* hydrogenase has a molecular mass of 89 kDa (subunits: 26 kDa and 63 kDa) and contains four redox centers: one nickel–iron center, one [3Fe4S], and two [4Fe-4S] clusters (Fig. 2A) [21, 25].

**Fig. 3:**
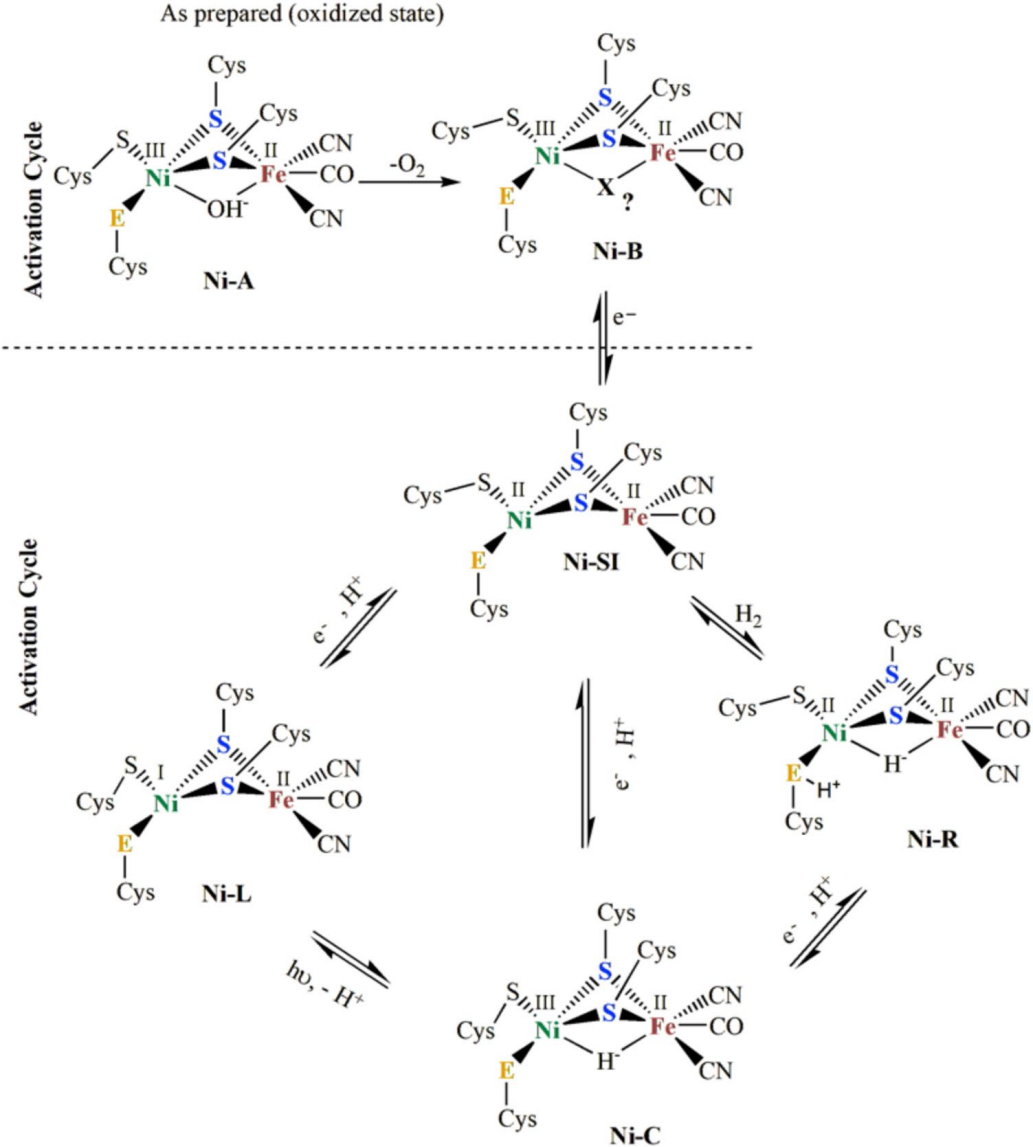
Different Oxidation states of [NiFe] Hydrogenase are involved in the activation and catalytic cycle. Adapted from [46]

EPR and Mössbauer (MB) spectroscopy have been the primary tools for studying the metal center composition of the enzyme, revealing the presence of four non-interacting centers: one [NiFe] center (where the Ni was assigned to an unusual trivalent oxidation state), one [3Fe4S] cluster (EPR-active), and two [4Fe4S] clusters (EPR-silent) [7, 16, 37, 38]. Intermediate redox species generated upon hydrogen interaction indicate that nickel is redox-active, cycling between trivalent and divalent oxidation states. These oxidation states are associated with redox-linked activation steps essential for full enzymatic activity. Three “faces” of the [NiFe] center have been identified in the isolated state as well as in the catalytic cycle by EPR: Ni-A (inactive), Ni-B (inactive-ready), and Ni-C (hydride bound), all of them in the formal + III state. Ni-C interacts with a proximal [4Fe4S] cluster. In addition, the oxidation state of nickel is formally + II in the case of Ni-Si_a_ (“nickel-silent-active”) [7, 16, 21, 23, 37–45], and Ni(I) in Ni-C light reacted (see below) (Fig. 3). Further redox state variations have been determined via the IR absorbance of the CO/CN^–^ ligands [46, 47]. The shifting of vibrational bands of CO/CN^–^ ligands between variable redox states arises from the alteration in electron density at their active sites. Indeed, the stretching vibration bands of νCO in Ni-A, Ni-B, Ni-SI, and Ni-C states for [NiFe] hydrogenases from *D. gigas* are observed at 1947 cm^−1^, 1946 cm^−1^, 1934 cm^−1^, and 1952 cm^−1^, respectively, suggesting different redox states in the entire catalytic cycle [46, 47].

### The isolated aerobic enzyme (resting)

**Fig. 4:**
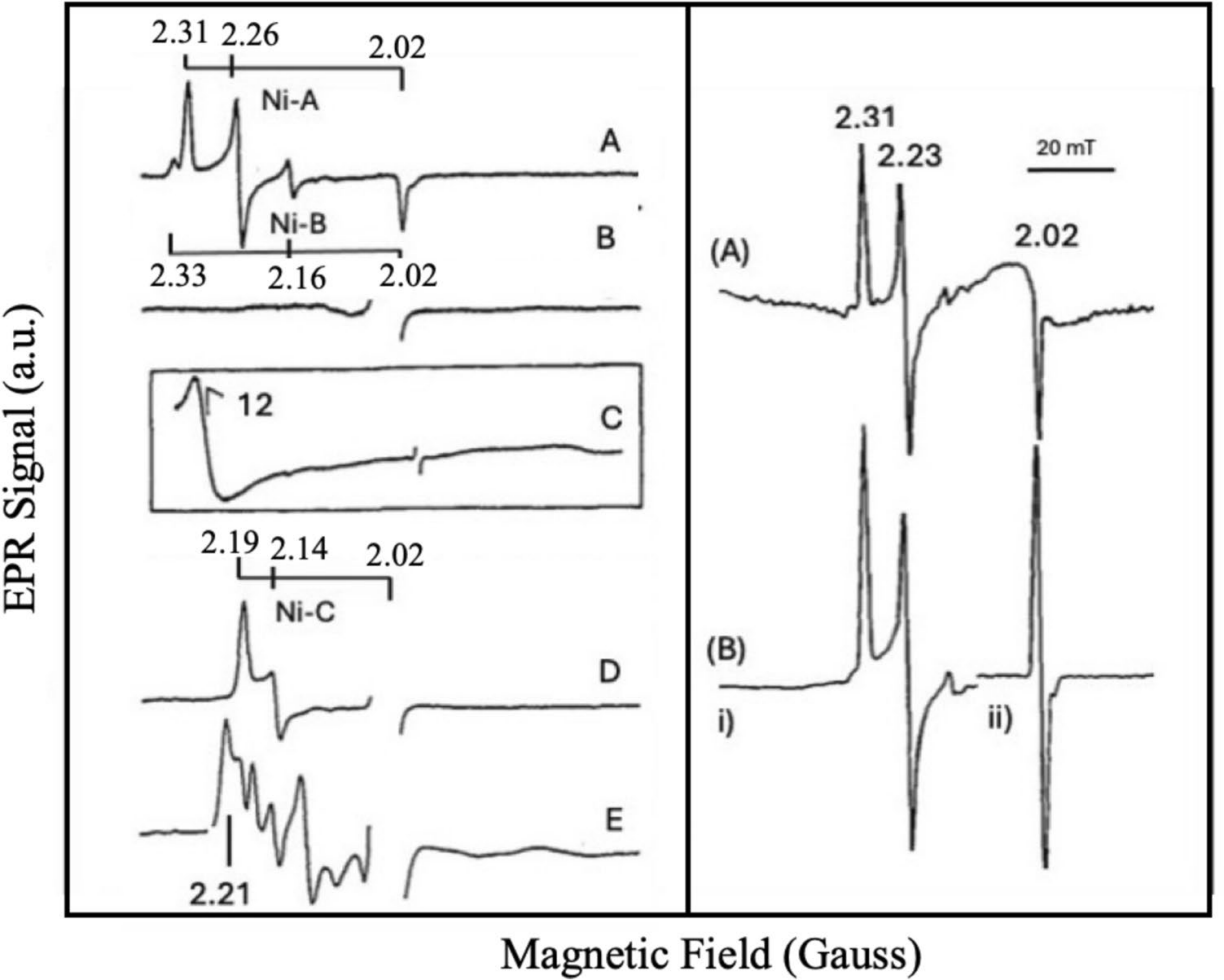
Representative spectra of *Desulfovibrio gigas* Hase (oxidized and H_2_ reacted). Left panel—**A** Native sample as isolated (oxidized state). Ni-A and Ni–B signals are observed at 77 K. (**B**) The so-called “EPR silent”, at 20 K. **C** The “g = 12” signal, “EPR silent” state as in **B**, but now observed in a wide field range at 4.2 K. **D** Ni-C signal at 20 K. **E** Same as **D**, but observed at 4.2 K. Right panel—Native sample as isolated. **A** Sample containing Ni-A signal as major species at 77 K. **B** Some as **A** but at 10 K. (i) Ni(III) features, (ii) g, 2.0 spectral region dominated by the [3Fe-4S]ox cluster. In addition, a schematic representation highlights the g-values associated with Ni-A, Ni-B and Ni-C. More details in the text. Adapted from [32, 51]

In the “aerobically as-isolated” enzyme, the nickel center is EPR-active, exhibiting two rhombic signals: a strong signal (Ni-signal A, Ni-A) with g-values at 2.31, 2.26, and 2.02, and a weaker signal (Ni-signal B, Ni–B) at 2.33, 2.16, and 2.02 [7, 16, 48, 49] (Fig. 4). Both signals remain observable up to 100 K without broadening. Ni-A and Ni-B states are key states in the initiation of the enzyme catalytic cycle, particularly in oxygen tolerance and reactivation processes. These states are oxidized forms of the active site and are distinguished based on their reactivity and spectroscopic signatures. Ni-A slowly reactivates under reducing conditions (termed the "unready" oxidized state), while Ni-B rapidly reactivates under reducing conditions (termed the "ready" oxidized state). More oxygen-tolerant hydrogenases tend to accumulate Ni-B rather than Ni-A [7, 16, 46–51].

Their relative intensities vary with protein preparation, and Ni-B can be enhanced by anaerobic redox cycling, suggesting to contain different oxygenated bound species. Isotopic substitution with (^61^Ni (I = 3/2) causes line broadening at g₁ = 2.31 and resolved hyperfine structures at g₂ = 2.23 (^61^A₂ = 1.5 mT) and g₃ = 2.02 (^61^A₃ = 2.7 mT), confirming that the paramagnetic nickel is at the origin of these signals [51]. This species is attributed to a low-spin Ni(III) center in a tetragonally distorted octahedral S = 1/2 system, with an unpaired electron in a dz^2^ orbital [32].

Combined EPR and Mössbauer spectroscopy definitively established an oxidized [3Fe4S] center (S = 1/2) (similar to the one isolated in *D. gigas* Ferredoxin II (FdII), a prototype of a [3Fe4S] cluster [52], with hyperfine broadening in EPR signals of ^57^Fe-enriched samples [13, 30]. The 4.2 K Mössbauer spectra of native and ^57^Fe-enriched samples revealed an intense quadrupole doublet, indicating two oxidized EPR-silent (S = 0) [4Fe4S]^+2^ clusters [30, 33]. These findings confirm that this hydrogenase contains four distinct redox centers: one nickel center, one [3Fe4S] cluster, and two [4Fe4S] clusters, which do not interact in their as-prepared state.

### Substrate-reacted enzyme – active state—magnetic interactions

#### *Structural reorganization of the Ni and [4Fe4S] centers upon reaction with H*_*2*_

##### (i) Ni signals

Upon hydrogen interaction, the g = 2.02 signal from the [3Fe4S]^+1^ center disappears first [30, 34] (mid-point redox potential − 70 mV (NHE), pH-independent), followed by the loss of Ni-signals A and B (Ni-A has a mid-point potential − 220 mV (NHE) and pH-dependent at 60 mV/pH unit), leading to a silent Ni(II) state [30–34], but concomitantly, a broad low-field signal (crossover at g≈12) emerges, resembling the “g = 12” signal attributed to a S = 2 spin state, similar to *D. gigas* ferredoxin II, which contains a single [3Fe4S] cluster [53]. (Fig. 4).

Mössbauer studies confirmed that, in fully reduced samples, the [3Fe4S] cluster remains reduced (S = 2) and does not convert to a [4Fe4S] cluster [33], indicating its presence in a catalytically active state. At this stage, the two [4Fe4S] centers are reduced to the + 1 state and remain spectroscopically distinct. After activation under hydrogen gas (or redox poising) restores a paramagnetic state of the nickel center, producing a transient rhombic EPR signal (Ni-C) with g₁ = 2.19, g₂ = 2.14, and g₃ = 2.02 appears but disappears with prolonged hydrogen exposure or excess sodium dithionite [31–33]. Isotopic substitution confirmed its assignment to Ni(III) (^61^A₃ = 2.0 mT) [54]. The Ni-C state is one of the most critical and well-studied intermediates in the catalytic cycle of [NiFe] hydrogenases and represents a key paramagnetic, reduced state involved in hydrogen activation. It is an active state in hydrogen catalysis and can be reduced further to Ni-R (fully reduced, Ni(II)), which releases hydrogen. It can be oxidized back to Ni-SI (inactive Ni(II)) under certain conditions. The Ni-C state forms when the hydrogen heterologous cleaves, a proton (H^+^) going to a nearby base and a hydride (H^–^) binding between Ni and Fe [7, 16, 21, 55]. The Ni-L site in light-activated [NiFe] hydrogenases is a crucial intermediate in the enzyme's catalytic cycle, particularly influenced by light [7, 16, 21, 56]. Ni-L (Light-sensitive state) is a reduced, paramagnetic (Ni(I)) state of the Ni–Fe active site. It is part of the catalytic cycle, occurring when the active Ni-SI state receives an electron. Ni-L is characterized by a low-spin Ni(I) center, stabilized by the Fe-S cluster. Ni-L is highly light-sensitive, and exposure to light can accelerate its conversion to the Ni-SI state (Ni(II)) (see Fig. 3). This photoconversion is due to energy absorption, which facilitates electron transfer and structural rearrangements at the active site. EPR (Electron Paramagnetic Resonance) spectroscopy identifies Ni-L as a Ni(I) species with distinct signals [7, 16, 21, 56, 57].

##### (ii) The nature of the ligands between Ni and Fe in different redox states of [NiFe] Hydrogenases.

The Ni-A (S = 1/2) is associated with the as-isolated (unready) form and features a nonprotein ligand (X) bridging the two metals. Ni-A and Ni-B states have been proposed to have a bridging hydroxide (OH^–^) ligand between Ni and Fe, having in Ni-B more flexible coordination environment (see Fig. 3). This ligand is proposed to be oxygenic (O^2–^ or OH^–^) in *Dg.*

Hase and an inorganic sulfide in *DvM*Hase. To determine its nature in *Dg*Hase, 35 GHz CW ^17^O ENDOR measurements were conducted with Ni-A containing major species exchanged into H_2_^17^O, the active Ni-C state (formed by H_2_-reduction of Ni-A in H_2_^17^O), and Ni-A regenerated by reoxidizing Ni-C in H_2_^17^O [39, 50, 58, 59].

In native Ni-A, the bridging ligand does not exchange with solvent. However, after a Ni-A → Ni-C → Ni-A cycle, an ^17^O label appears at the active site, as confirmed by ENDOR. The substantial isotropic component (*a*_iso_(^17^O) ≈ 11 MHz) confirms that the solvent-derived ^17^O ligates Ni, identifying X in Ni-A as an oxygenic species. This aligns with prior EPR results suggesting the same for Ni-B [60].

The small ^57^Fe hyperfine coupling observed in Ni-A (A(^57^Fe) ~ 0.9 MHz) persists in Ni-C (A(^57^Fe) ~ 0.8 MHz), but the ^17^O signal vanishes upon reduction to Ni-C and reappears upon reoxidation to Ni-A. Analysis of the electronic structure suggests that the oxygenic bridge in Ni-A(B) is lost in Ni-C and reforms from solvent upon reoxidation. This implies that Ni-C formation opens Ni/Fe coordination sites, potentially playing a key role in enzyme activity [50, 58–60].

##### (iii) Ni site and magnetic interactions.

At low temperatures (< 10 K), another complex g = 2.21 EPR signal emerges at redox levels around Ni-C formation [32, 61]. The observation of this signal requires high microwave power, indicating a fast-relaxing species. A heterolytic hydrogen cleavage mechanism has been proposed in bacterial hydrogenases [59]. Ni-C was initially attributed to a nickel-hydride species [31, 32], but the relaxation behavior of the g = 2.21 signal suggests spin–spin interactions rather than a simple paramagnet (see Fig. 4). It was further interpreted as a split Ni-C signal due to interaction with a [4Fe4S]^+1^ cluster [61].

Supporting this assignment was the study of redox states of the enzyme prepared, with the [4Fe4S] clusters either diamagnetic or paramagnetic. In the paramagnetic state, magnetic coupling between metal centers induces a complex low-temperature EPR spectrum, a split Ni-C signal, and increased Ni center relaxation rates. Simulations of the split Ni-C signal at X-, Q-, and S-band frequencies, using a point dipole model for dipolar and exchange interactions, confirm that only one [4Fe4S]^+1^ cluster is significantly coupled to the Ni site [63]. This provided a detailed description of their relative arrangement, by spin–spin and spin–lattice relaxation times were measured in both redox states using power saturation, pulsed EPR at low temperatures, and EPR line broadening studies at higher temperatures [63].

## Mononuclear molybdenum enzymes of the xanthine oxidase family

**Fig. 5:**
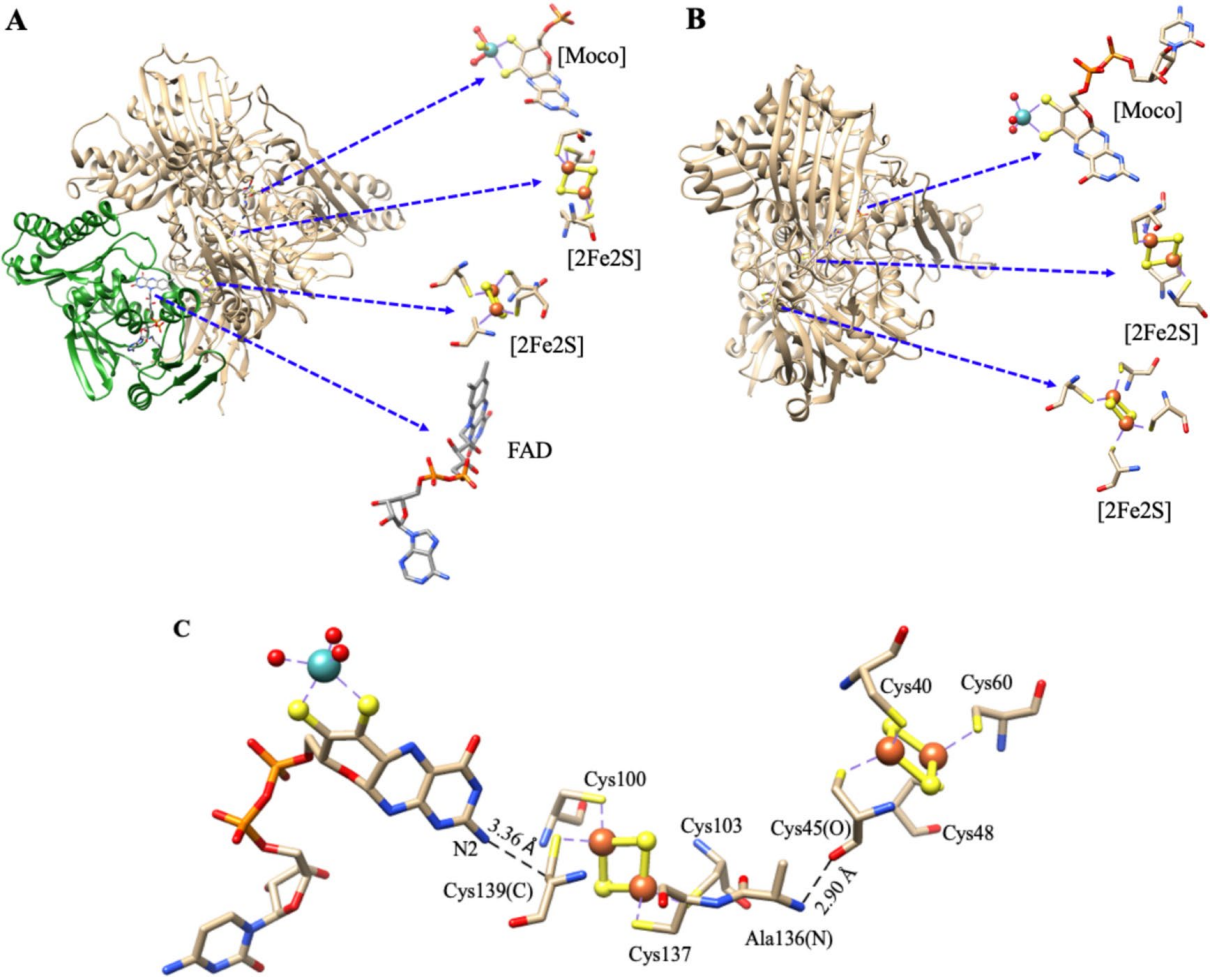
3D structures of Xanthine oxidase (PDB: 1FIQ, bovine milk) (**A**) and Aldehyde oxidoreductase (PDB: 1VLB, *D. gigas*) (**B**). XO contains four redox centers: a molybdenum cofactor (MoCo, molybdopterin cytosine dinucleotide), two [2Fe2S] centers, and an FAD moiety. AOR shares strong structural similarities with XO but lacks the FAD-binding domain (green) found in XO. **C** The relative arrangement and interaction of three cofactors in AOR, molybdopterin cytosine dinucleotide, [2Fe2S] center type I, and [2Fe2S] center type II are shown by the X-ray crystal structure of AOR, and show a possible electron-transfer chain between three cofactors. Mo is shown as a cyan sphere, Fe as larger red spheres, S in yellow, and O in small red. Adapted from [69, 70]

Aldehyde oxidoreductases (AORs) from various *Desulfovibrio* species—*D. gigas*, *D. alaskensis*, *D. aminophilus*, and *D. desulfuricans* are homologous enzymes within the molybdenum hydroxylase family. These enzymes catalyze the hydroxylation of aldehydes to carboxylic acids [8, 64–68]. As members of the xanthine oxidase (XO) enzyme family, these molybdoenzymes also contain two [2Fe2S] clusters, designated Fe/S I and Fe/S II, coordinated by distinct cysteine motifs (Fig. 5A and B). These clusters facilitate electron transfer from the substrate to an acceptor, such as an FAD moiety (as in XO) or an external electron-transferring protein [8, 61–68].

X-ray crystallography has shown that in *D. gigas* AOR (*Dg*AOR), the Fe/S I cluster is coordinated via a ferredoxin-type motif near the protein surface, while Fe/S II is ligated by an atypical cysteine motif and is in direct contact with the molybdopterin cofactor (Fig. 5B) [69, 70]. Relative distances between three cofactors in MOP, molybdopterin cytosine dinucleotide, [2Fe2S] center type I, and [2Fe2S] center type II are shown in Fig. 5C. The inter-connection between these redox cofactors is proposed as an electron-transfer pathway involving electron relay from one cofactor to another [69, 70]. The spectroscopic and crystallographic available data for *D. alaskensis*, *D. gigas,* and *D. desulfuricans* AORs support that both the spatial orientation and links among paramagnetic cofactors are conserved in this group of enzymes. The cluster spatial arrangement is illustrated in Fig. 5 from the gene sequence and crystal structure of the *Dd*AOR.

**Fig. 6:**
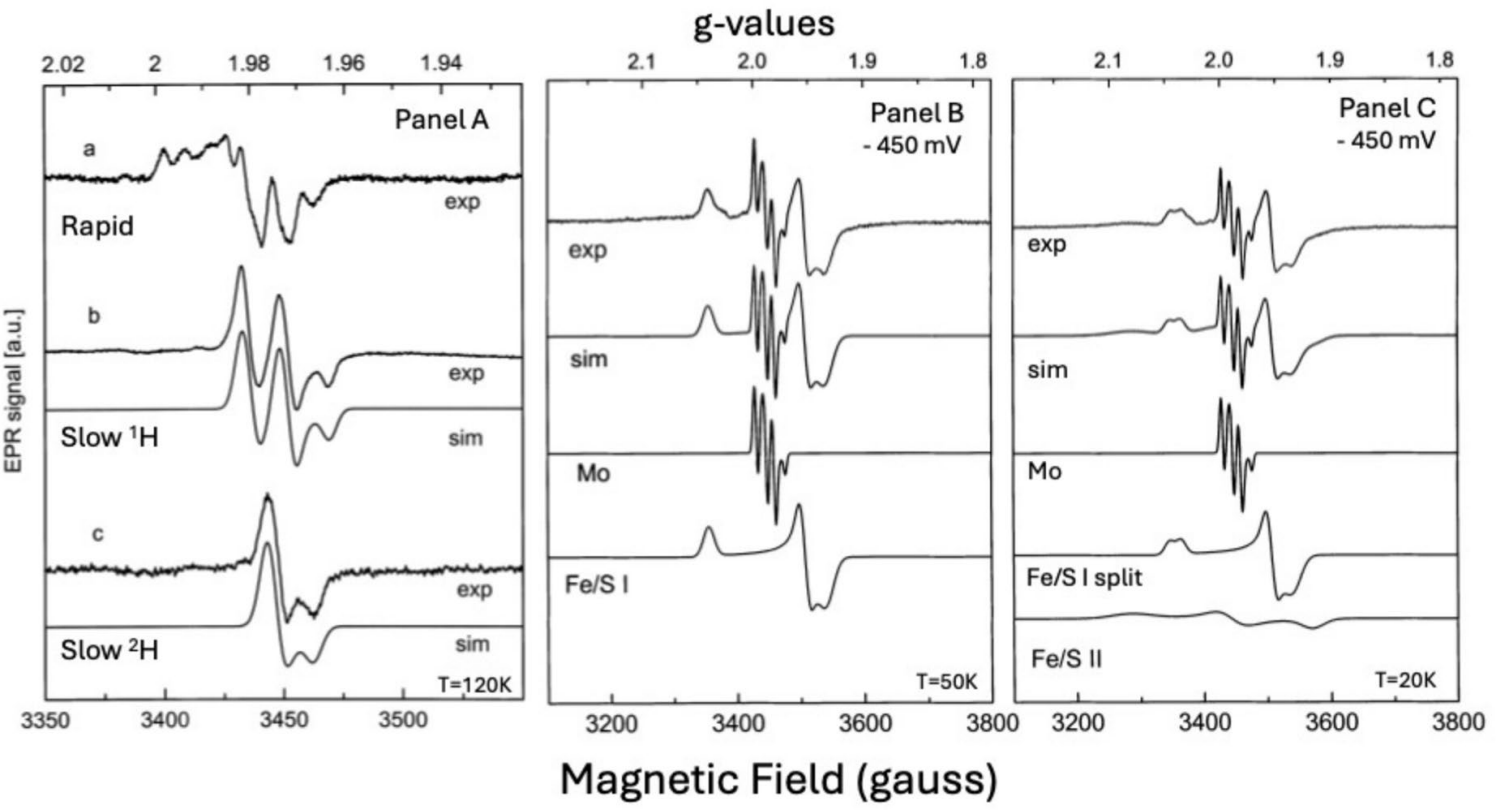
EPR spectra of *D. alaskensis* AOR. **Panel A**—Mo(V) signals (a) Rapid-type signal obtained in as-isolated samples treated with benzaldehyde. (b) Slow-type signal obtained in samples after 10 min reaction with excess dithionite. (c) as (b) but in a deuterated sample. Experimental conditions: temperature, 120 K; microwave power, 2 mW; modulation amplitude, 4 G_pp_; microwave frequency, 9.5 GHz. **Panel B**—samples at − 450 mV. Experimental conditions: temperature, 50 K; microwave power, 0.2 mW; modulation amplitude, 4 G_pp_; microwave frequency, 9.5 GHz. **Panel C**—Experimental conditions: as in (**A**) except the temperature (20 K) and microwave power (0.06 mW). Simulations were performed as indicated in (ref). Adapted from [68]

Figure 6 shows EPR spectra for *D. alaskensis* AOR under various conditions (oxidation states and temperatures), which probe magnetic interactions between the Mo site, nearby protons, and the Fe/S I cluster, as well as between Fe/S I and Fe/S II [68]. Two types of Mo(V)-associated EPR signals were identified: slow-type that develops after prolonged dithionite reduction, exhibiting nearly axial features with hyperfine splitting from a single proton (I = 1/2), confirmed using deuterium exchanged samples (Fig. 6-panel B, C) and rapid-type observed after benzaldehyde reduction, resembling a Michaelis complex analogue, also with proton hyperfine interactions (Fig. 6-panel A), simulation included for deuterated and non-deuterated samples [68].

Additionally, spectroscopic detected splittings suggest super-exchange coupling between the Mo site and adjacent Fe/S clusters. Low-temperature (< 70 K) EPR spectra reveal two [2Fe2S] clusters: Fe/S I, with narrower linewidths visible at 50 K, and Fe/S II, with broader signals at 20 K. Redox poised samples demonstrate temperature-dependent magnetic and super-exchange interactions between Mo(V) and the proximal Fe/S I center, evidenced by isotropic splittings and enhanced relaxation [68, 71, 72]. Dipolar interactions dominate between Fe/S I and Fe/S II (anisotropic splitting) (see Fig. 6-panels B and C). It is observed within this AOR class that the Fe/S I signal is fully split when the Fe/S II is fully sharpened. This fact, together with a similar spin concentration value for the two cofactors (detected in samples reduced with sodium dithionite at − 450 mV), indicates that reduction of Fe/S II produces magnetic coupling of the Fe/S I signal [68].

The magnitudes and type of splitting observed in *D. alaskensis* AOR are similar to those observed in the *D. gigas* [73] and *D. desulfuricans* [74] proteins, indicating that both the spatial orientation and links among paramagnetic cofactors are conserved in both AOR enzymes.

High-resolution structural data from *Dg*AOR enabled us to explore more deeply the magnetic interactions among its redox centers and to assign structurally the electronic pathways. Isotropic exchange coupling between the Mo center and the proximal Fe/S cluster was studied using native and polyalcohol-inhibited samples. EPR and QM/MM studies [17] modelled the Mo site as weakly coupled paramagnetic centers with distinct relaxation behavior [75–78].

The Fe/S I and Fe/S II centers could be distinguished by their g-values, temperature effects, redox potentials, and reduction rates [79, 80], in parallel to the results observed for *D. alaskensis* AOR. Their EPR signatures differ: Signal I (Fe/S I), less anisotropic, typical of ferredoxin-type signals (g_av_ ~ 1.96) and Signal II (Fe/S II) with broader spectral lines, higher g-values, and rapid relaxation properties attributed to low-lying excited states and double exchange interactions.

Spectroscopic and structural assignment of the two [2Fe2S]^+1^ clusters was aided by analyzing the Mo(V) EPR signal, as a probe [75, 81–85]. When all three metal centers are paramagnetic, spin–spin interactions cause spectral splitting at low temperatures. Modelling these based on the crystal structure allowed identification of the reducible [2Fe2S] clusters. Temperature-dependent behavior supports that Mo(V) couples magnetically with the cluster responsible for signal II—establishing this as the cluster nearest the Mo cofactor in both XO and *Dg*AOR [75, 81–85].

Combining crystallographic and EPR data enables a straightforward method to determine the relative positions of Fe/S clusters with respect to the Mo site. These spectroscopic insights are broadly applicable across the XO enzyme family, which consistently features two [2Fe2S] clusters and a Moco (molybdopterin cytosine dinucleotide or molybdopterin). In *Dg*AOR, the protein domain organization and cofactor environment define a buried, mechanistically distinctive catalytic site. The metal coordination geometries are well-resolved, and the proximity of redox centers (~ 12 Å) favors efficient intramolecular electron transfer during the catalytic cycle, mediated by the pterin cofactor [69, 70]. Spectroscopic evidence suggests that cofactor distances and orientations are preserved among all the homologs *Desulfovibrio sp.* AORs [69, 70].

From an evolutionary perspective, the 3D structures and cofactor arrangement in these enzymes are remarkable. The flavin domain serves as the terminal electron sink in XO and CO dehydrogenase ([MoCu]), see below [65, 86]. In *D. gigas* AOR, an external flavoprotein may fulfil this role [70]. A similar cofactor arrangement was recently proposed for CODH ([MoCu]), lacking its flavin domain [65, 70, 86]. Structurally, XO is a dimer of a single 300 kDa polypeptide with four cofactors [65]. CODH dehydrogenase is a dimer of three subunits (88 kDa molybdopterin, 30.2 kDa flavoprotein, 17.2 kDa Fe/S protein), sharing structural features with XO [62, 86]. AORs, in contrast, are dimers of shorter polypeptides lacking the flavin domain present in XO and CODH ([MoCu]). The Mo and Fe/S subunits of CODH ([MoCu]) are nearly superimposable on *D. gigas* AOR (70,86).

## Carbon-monoxide dehydrogenase (CODH)

Carbon monoxide (CO) poisons many organisms, but particular microorganisms (both anaerobic and aerobic soil bacteria) have special metalloenzymes, namely carbon-monoxide dehydrogenase (CODH), which can use CO productively as the source of carbon and energy [5, 10, 11, 87, 88]. CODHs are classified into two classes based on their active sites and O_2_ sensitivity [89]. The first class is O_2_-sensitive CODHs harbor heterometallic [NiFeS] cofactor, which catalyzes the reversible redox reaction (both directions), CO + H_2_O $\leftrightarrow$ CO_2_ + 2e^−^  + 2H^+^ [11, 87] with turnover frequency (TOF) of 40,000 s^–1^ for CO oxidation and 45 s^–1^ for CO_2_ reduction [90]. The second class is non-O_2_-sensitive CODHs harbouring heterometallic [MoCu] cofactor, which catalyzes the same redox reaction, in only one direction (oxidation direction) with a moderate TOF of 100 s^–1^ [86, 88].

### [MoCu] CODH

**Fig. 7:**
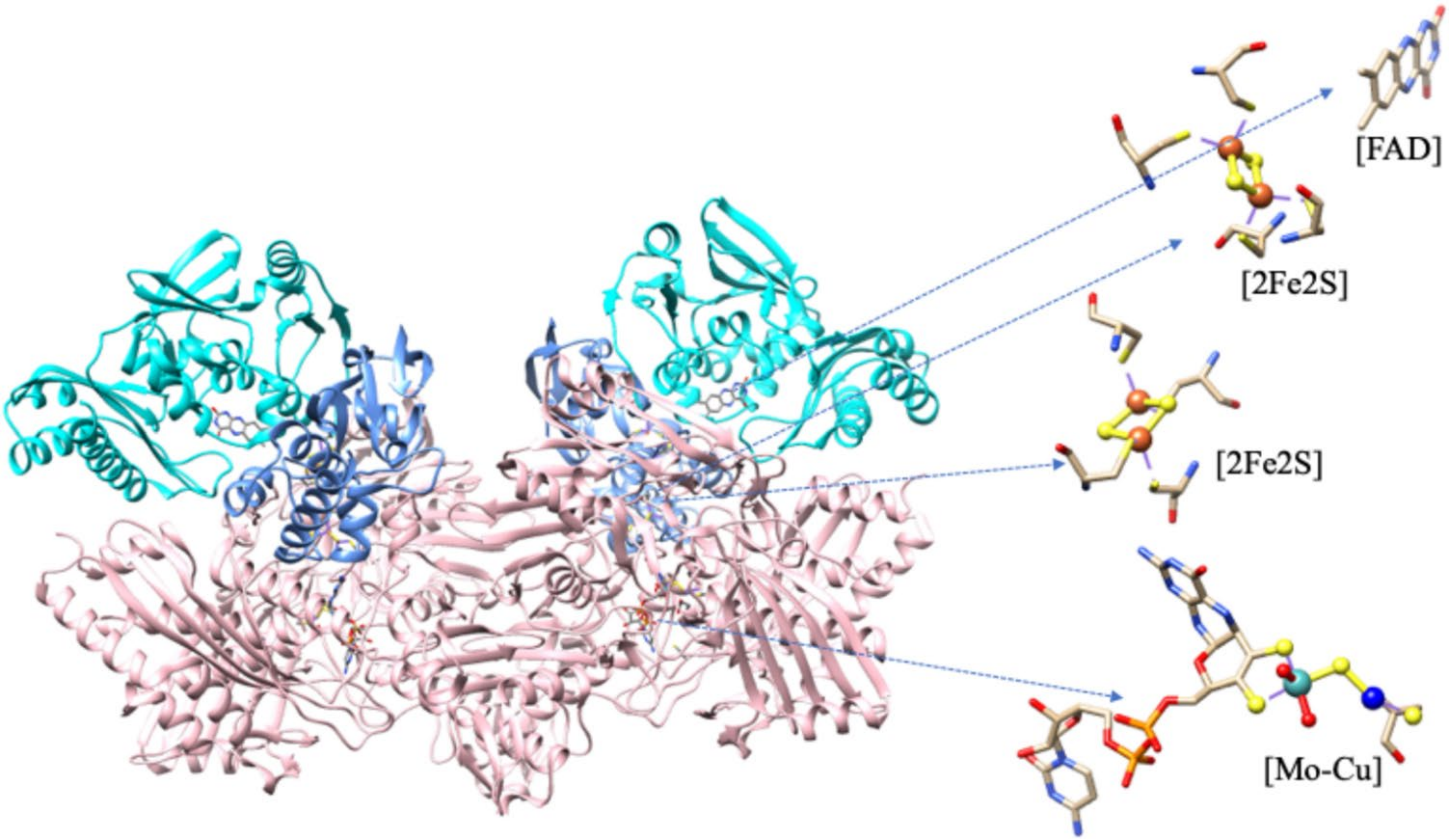
Crystal structure of the dimer of trimer of aerobic [MoCu]-CODH from *O. carboxidovorans* (PDB: 1N5W): each trimer contains three subunits, including L-subunit; molybdo-protein (pink), S-subunit; Fe-S protein (blue) and M subunit; flavoprotein (cyan). Highlighted the [MoCu] active site, two [2Fe2S] clusters and FAD with distances among these redox components: Mo (cyan ball), Cu (blue ball), Fe (bigger red ball) S (yellow ball) and O (small red ball)

The [MoCu] CODH belongs to the xanthine oxidase family. The crystal structure reveals that the protein is a dimer of a heterotrimer with 277 kDa [MoCu] containing iron-sulfur flavoprotein [86, 90–92]. Each heterotrimer is composed of an 88.7 kDa molybdoprotein (Large subunit (L)) containing the active site of CODH, a 30.2 kDa flavoprotein (Medium subunit (M)) containing FAD cofactor, and a 17.8 kDa iron-sulfur protein (small subunit (S)) containing two types of [2Fe2S] clusters (Fig. 7) [86, 90–92]. Its active site harbors an unprecedented heterobimetallic cluster composed of Cu and Mo; a bridging μ_2_-S ligand connects these two metals. In the catalytic cycle, the fully oxidized state of Mo(VI) in the bimetallic center (Mo(VI)Cu(I)) of the [MoCu] CODH catalyzes the two-electron oxidation of CO to CO_2_ with concomitant two-electron reduction of Mo(VI) to Mo(IV) (Mo(IV)Cu(I)). Therefore, during catalysis, Mo(VI) accepts electrons from the CO substrate to reduce to Mo(IV), and Cu(I) is capable of binding the CO substrate, but it remains same oxidation state due to a high degree of delocalization in the Mo-S-Cu unit [91, 93].

However, the detailed mechanism is still under debate [93–97]. During the re-oxidation of Mo^IV^ to Mo^VI^, the Mo^V^ species may be generated from Mo(IV) by transferring one electron to a Fe/S cluster prior to completing the catalytic cycle. These cofactors establish an intramolecular electron transport that delivers the electrons generated through the oxidation of CO at the [CuSMoO_2_] cluster to [2Fe2S] I, [2Fe2S] II, and finally to FAD, from where they are fed into a CO-insensitive respiratory chain to generate a membrane potential [98, 99]. The functional [MoCu] CODH is EPR silent, suggesting Cu(I) state, but inactive [MoCu] CODH shows a small Mo(V) signal like XOR, indicating loss of copper-sulfur bridge [99, 100]. In the catalytic cycle, the re-oxidation of Mo(IV) to Mo(VI) via Mo(V) by donating one electron to Fe–S cluster prior to complete the catalytic cycle. The Mo(V) shows a rhombic EPR signal with g_xyz_ (2.0010, 1.9604, 1.9549) and strong hyperfine coupling with Cu-nuclei (I = 3/2), A_xyz_ (117, 164, 132 MHz), indicating the high degree of electronic communication between Mo and Cu through the S-bridge [12, 86]. Interestingly, the EPR spectrum of active CODH enzyme disappears when it is treated with potassium cyanide and displays a new rhombic EPR signal at g_xyz_ = 1.977, 1.967, and 1.953, suggesting Cu affects the magnetic state of the Mo ion [86]. Moreover, Cu can be replaced by Ag in Mo/Cu-CODH under suitable conditions. When Cu(I) is replaced by Ag(I), the enzyme also shows Mo(V) EPR signal, with g_xyz_ (2.0043, 1.9595, 1.9540) and strong hyperfine coupling with Ag-nuclei (I = ½) with A_xyz_ (82, 79, 82 MHz) upon reduction with CO [5, 101]. The silver-substituted enzyme retains the catalytic activity of the native enzyme, with a slower rate constant compared to the native enzyme [101].

### [NiFe] carbon monoxide dehydrogenases

The [NiFe] carbon monoxide dehydrogenases ([NiFe] CODH) catalyze the reversible interconversion of CO with H_2_O to CO_2_ with 2H^+^ and 2e^−^ [6, 11, 102]. The 3D structures of [NiFe]-CODH from *Carboxydothermus hydrogenoformans* (CODH_Ch_), *Moorella thermoacetica* (CODH_*Mt*_), and *Rhodospirillum rubrum* (CODH_*Rr*_) have been reported [103–107]. These organisms have the same protein structure, but have two different types of active site structures: [Ni4Fe5S] active center in CODH_*Ch*_ and [Ni4Fe4S] active centre in CODH_*Rr*_ and CODH_*Mt*_.

**Fig. 8:**
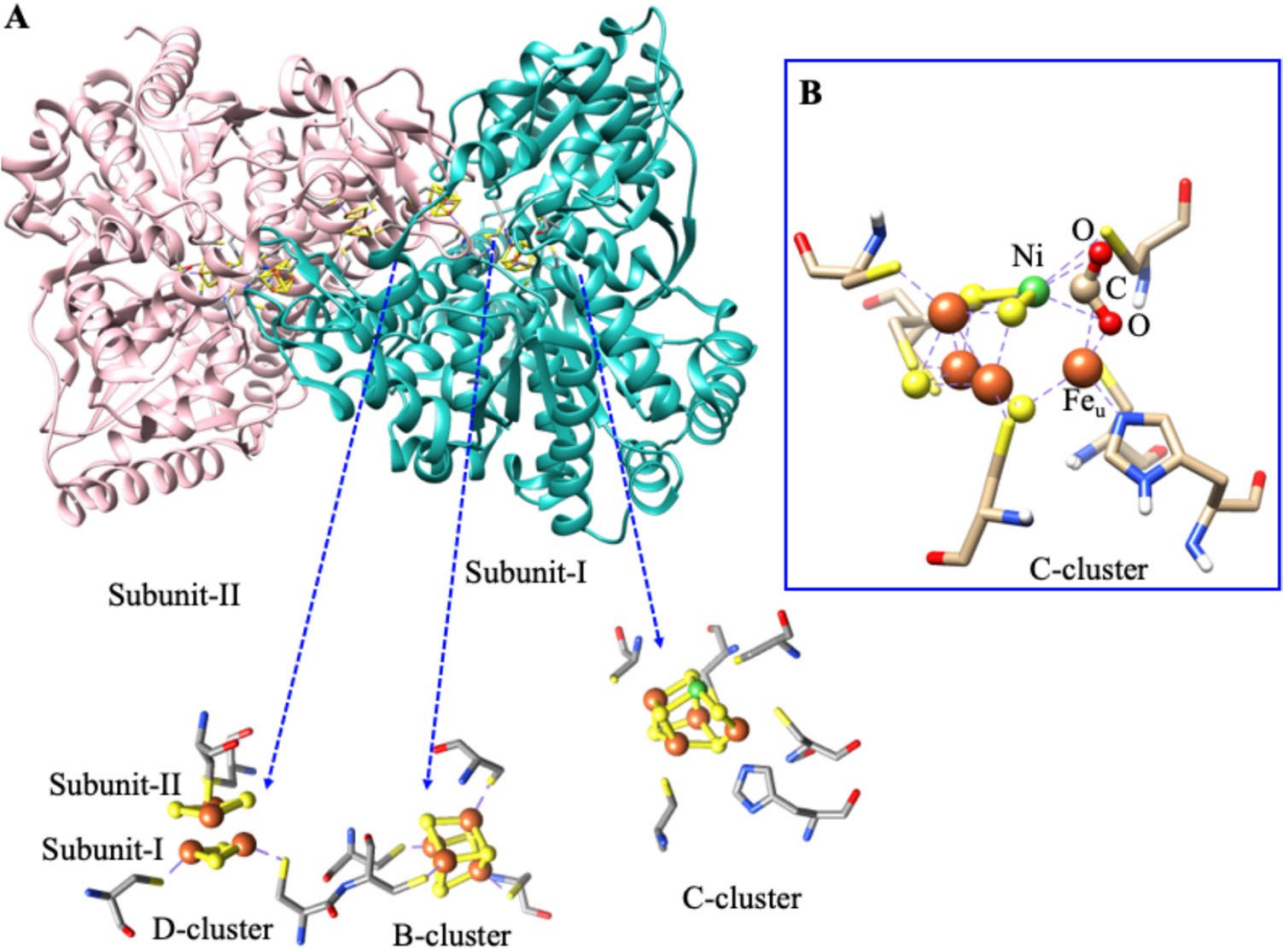
**A** The Crystal structure of the [NiFe]-CODH homodimer (PDB:1SU6) from *C. hydrogenoformans and t*he metal-sulfur clusters are highlighted. **B** Crystal structure of the C-cluster of CO_2_ bound [NiFe]-CODH (PDB: 4UDX) from *C. hydrogenoformans*. Fe (red ball); Ni (green ball), and S (yellow ball)

The crystal structure of [NiFe]-CODH from *C. hydrogenoformans* is shown in Fig. 8. The [NiFe]-CODH is a large (molecular mass of 130 kDa) and complex homodimeric protein [104]. Each subunit harbors one conventional cubane-[4Fe4S] cluster (B-cluster), one asymmetrical [Ni4Fe5S] cluster (C-cluster), and an additional half part of the cubane [4Fe4S] cluster (D-cluster) at the interface of the dimer (Fig. 8A). Among them, the C-cluster is a catalytic centre where reversible reduction of CO_2_ occurs [108, 109]. The C-cluster, [Ni4Fe5S], is an unusual structure where one Ni atom, three Fe atoms, and four inorganic S form a cuboidal-like [Ni3Fe4S] cluster, and one additional Fe atom (unique Fe or Feu) is extraneous to the [Ni3Fe4S] inserting at the Ni–S edge [103] and subsequent studies have demonstrated CO_2_-bound C-cluster, [Ni4Fe4S(CO_2_)] in which CO_2_ is inserted between Ni and Feu (Fig. 8B) [110, 111].

The C-cluster has four oxidation states: a fully oxidized inactive state (C_ox_), a one-electron reduced active state (C_red1_), a two-electron reduced intermediate state (C_int_), and a three-electron reduced active state (C_red2_). C_ox_ and C_int_ both are EPR silent with spin state S = 0, whereas C_red1_ and C_red2_ both are EPR active, having spin states S = ½ and exhibiting EPR signals with g_1,2,3_ = 2.01, 1.81, and 1.65 and g_1,2,3_ = 1.97, 1.87, and 1.75, respectively [6, 102, 108, 112, 113]. Both states, C_red1_ and C_red2,_ have similar EPR data, suggesting the unchanged electronic structure of [3Fe4S] fragment. The Ni K- and L-edge XAS studies described the low-spin EPR-silent Ni(II) for both C_red1_ and C_red2_ [114–116]. The absence of ^61^Ni hyperfine coupling in the C_red1_ EPR signal suggests that electronically decoupled from the cluster and does not contribute to the spin-coupling mechanism [116]. The Mössbauer spectrum of C_ox_ shows a typical [4Fe4S]^2+^ with no proof of Fe in Fe_u_ [102]. Mössbauer spectra of the C_red1_ state [112] describe high-spin Fe(II), Fe(III) oxidation states for [3Fe4S] fragment and high-spin Fe(II) oxidation state for Feu (unique Fe) [6, 102, 108].

The oxidation of CO involves 1e^−^ reductive activation of inactive C_ox_ to active C_red1_ at below potentials of − 100 mV, followed by 2e^−^ reduction to yield C_red2_ at below potential of − 500 mV, close to the potential of the CO/CO_2_ redox couple [6, 102, 116].

The C_red1_, C_int_, and C_red2_ states are involved in the catalytic cycle. All proposed mechanisms generally presume that the CO₂-bound [Ni–Fe]-CODH structure represents a catalytic intermediate where the C atom of CO_2_ is coordinated to Ni, one O atom of CO_2_ is coordinated to Feu (Fig. 8B) [6, 102]. Based on the structure, the CO₂ binding to C_red2_ is thought to take place through a dissociative mechanism, yielding Ni-CO and Fe_u_-OH, suggesting strong magnetic interactions between Ni and Fe_u._ This interaction disturbs the EPR g‑values of C_red1_, indicating the key role of spin coupling between the Ni center and the FeS cluster in the redox regulation of CO₂ binding and release [6, 102, 116].

## Magnetic interactions and enzymatic function in Ni, Mo, Cu-containing complex enzymes

Magnetic interactions in metal enzymes are direct manifestations of unpaired electron spins residing in transition metal centers or iron-sulfur clusters. These interactions are tightly correlated with redox chemistry, substrate binding, catalytic turnover, and electron transfer (ET) dynamics, all of which are fundamental to enzymatic activity. EPR and related spectroscopic tools proved as essential for studying these interactions and to characterize electron transfer processes in catalytically active redox states and electron transfer sites.

### [NiFe]-hydrogenases

The [NiFe] are bimetallic active sites are coupled with iron-sulfur (Fe/S) clusters that serve as electron relays [41]. In catalytically relevant states (e.g., Ni-C, Ni-L), magnetic interactions between unpaired d-electrons and surrounding nuclei (such as protons or Fe/S centers) can be observed by EPR and ENDOR [50–60]. The magnetic coupling between the metal center and nearby [4Fe4S] clusters controls electron flux to and from the active site, directly modulating H_2_ production or oxidation [50–60]. Subtle changes in hyperfine couplings reflect protonation events, which are part of the proton-coupled electron transfer (PCET) mechanism central to catalysis [46, 62]. Indeed, the Fe in the [NiFe] active site is primarily redox inactive, but shows hyperfine couplings influenced by the nature of its ligands, like CO and CN⁻. Ligand substitution impacts on the Fe-centre, affecting magnetic coupling to the Ni center [46, 47]. In addition, EPR values of variable redox states of the Ni–C (H-bound) and Ni–L (light-induced) reveal significant changes in Ni-hyperfine coupling due to alterations in spin density and ligand binding [31–33, 54]. These couplings may provide critical insights into the electronic and geometric environment of the active site, which can lead to the design of synthetic catalysts that efficiently activate hydrogen.

### Mo containing enzymes (xanthine oxidase family)

These enzymes contain mononuclear Mo centers coordinated by sulfur ligands and dithiolene cofactors (like MPT) [64]. During catalysis, Mo cycles between Mo(VI), Mo(V), and Mo(IV) oxidation states. The Mo(V) intermediate is EPR-active and often stabilized by spin coupling with surrounding centers (e.g., FeS clusters or Cu centers in certain oxidases). Magnetic interactions in Mo(V) states reveal the electronic structure and geometry of the active site, giving insights into substrate binding orientation and electron delocalization [67, 68, 72]. In enzymes like aldehyde oxidase or xanthine oxidase, the strength of magnetic interactions often correlates with the efficiency of oxidative transformations. Indeed, several inhibitors of the XO.

family, such as arsenite, formaldehyde, glycerol and ethylene glycol interact with the Mo-site to yield Mo(V)-inhibitor complex, which changes in the electronic structure of the Mo(V) site, favoring a larger magnetic coupling with the proximal FeS center compared to dithionite-reduced *Dg*AOR [76, 117]. These inhibited enzyme states are vital for understanding changes in the integrity of the electron transfer chain upon inhibition and providing insights that can guide the rational design of drugs.

### Carbon monoxide dehydrogenase (CODH)

Generally, CODHs are two classes: [MoCu]-CODHs and [NiFe]-CODHs. [MoCu]-CODH belongs to the xanthine oxidase family, containing an unprecedented heterobimetallic cluster, [Mo–S–Cu] cluster. In catalytic cycle, Mo(V) is generated and shows a rhombic EPR signal with strong hyperfine coupling to Cu-nuclei [101] and even Cu substituted Ag-Mo-CODH shows Mo(V) EPR signal with strong hyperfine coupling to Ag-nuclei, suggesting that CO binds Cu-site, triggering electron density to Mo-site for activation of CO across the Mo–S–Cu motif [101]. Moreover, the Ag-substituted CODH exhibits a slow enzymatic activity, indicating that substitution of the native metal by other non-native metals alters the electron flow in the catalytic pathway, providing valuable insights to design artificial catalysts that can modulate the activity.

The other class of CODH is [NiFe]-CODH C-cluster containing [Ni4Fe5S] and CO_2_-bound C-cluster, [Ni4Fe4S(CO_2_)], which represents a catalytic intermediate [6, 102]. The CO₂ binding C-cluster significantly changes g-values, suggesting that substrate binding modulates the magnetic interactions between Ni and Fe centers and therefore, the CO₂-bound C-cluster state leads to the cleavage of the C–O bond in CO₂, yielding Ni–CO and Fe_u_–OH species [116, 118]. This CO_2_-bound intermediate provides a blueprint for designing catalysts that can efficiently activate CO and CO₂.

EPR spectroscopy reveals redox transitions involving Ni(II)/Ni(I), coupled with Fe-S oxidation changes. The magnetic interaction between Ni and Fe/S centers enables rapid electron redistribution, essential for breaking the strong C≡O bond in CO. In CODH/ACS (acetyl-CoA synthase complex), inter-cluster magnetic coupling (between the C-cluster and the A-cluster) aligns with conformational changes that facilitate substrate channelling and catalysis [112, 119].

### Final remarks

Although out of the scope of this review, MoFe Nitrogenases [1, 120] and Copper Enzymes (LPMO (lytic polysaccharide monooxygenases), Azurin, Cytochrome c Oxidase) [10, 121] are also case studies of this type of interactions. The MoFe protein contains the FeMo-cofactor ([FeMoSC]) at the active site. It is electronically complex and paramagnetic in several redox states (e.g., E_0,_ E_4_). Magnetic interactions are crucial in stabilizing and identifying intermediates during the 8-electron reduction of N_2_ to NH_3_ [122].

The E4 “Janus intermediate”, containing two bridging hydrides, shows a unique EPR signature reflecting spin delocalization across multiple metal centers. Spin-coupled Fe/S clusters (P-cluster, Fe protein) deliver electrons sequentially—these steps are regulated by precise magnetic interactions that affect timing and directionality of ET. The EPR, ENDOR, and Mössbauer studies of nitrogenase show how the magnetic structure controls substrate binding, activation, and releasing [1, 120, 123–129].

Mononuclear Cu(II) centers, such as LPMO, Azurin, and Cytochrome c Oxidase are often EPR-active, and magnetic anisotropy (g-tensor) gives detailed information about geometry and ligand field. In LPMOs Cu(II)/Cu(I) cycling, driven by reductants and oxygen, is tightly linked to magnetic properties observed in EPR. Magnetic coupling between Cu and adjacent redox cofactors (like tyrosyl radicals or Fe centers in multi-domain oxidases) can modulate the redox potential and catalytic cycle [10, 121, 130–132].

## Conclusions

Magnetic interactions between Fe/S clusters and other metal centers, such as nickel and molybdenum, play a pivotal role in defining the catalytic efficiency, redox properties, and structural stability of key metalloenzymes. The intricate electronic coupling and spin exchange mechanisms modulate enzymatic function.

In all these enzymes, magnetic interactions are not peripheral—they reflect and influence the core chemical transformations taking place. These interactions: (i) serve as spectroscopic fingerprints for redox intermediates, (ii) indicate electron transfer pathways and their directionality, and (iii) provide mechanistic insight into substrate binding, bond cleavage, and product release, influencing the thermodynamic and kinetic parameters of catalysis.

Understanding these magnetic features is an interesting and stimulating theoretical problem, but also directly informs enzyme engineering, biocatalyst design, and the development of biomimetic catalysts for energy conversion and environmental remediation.

Advances in spectroscopic techniques, including electron paramagnetic resonance and Mössbauer spectroscopy, along with computational modelling, have significantly deepened our understanding of these interactions at the molecular level.

By elucidating the fundamental principles governing these magnetic interactions, insights may contribute not only to the field of bioinorganic chemistry but also to the rational design of biomimetic catalysts with potential applications in renewable energy, biotechnology, and industrial catalysis. Future research integrating experimental and theoretical approaches will further refine our comprehension of metalloenzyme function, paving the way for innovative strategies in enzyme engineering and sustainable catalysis.

## Data Availability

No datasets were generated or analysed during the current study.

## Abbreviations

AOR: Aldehyde oxidoreductase
CODH: Carbon monoxide dehydrogenase
Ch: *Carboxydothermus hydrogenoformans*
Da: *Desulfovibrio alaskensis*
Dd: *Desulfivibrio desulfuricans* ATCC2774
Dg: *Desulfovibrio gigas*
DgAOR: *Desulfovibrio gigas* Aldehyde oxidoreductase
DgHase: *Desulfovibrio gigas* Hydrogenase
DvM: *Desulfovibrio vulgaris* Myasaki
DvMHase: *Desulfovibrio vulgaris* Myasaki hydrogenase
ENDOR: Electron-nuclear double resonance
EPR: Electron Paramagnetic Resonance
ET: Electron transfer
FAD: Flavin adenine dinucleotide
Fd: Ferredoxin
Fe/S: Iron-sulfur cluster
Feu: Unique iron
Hase: Hydrogenase
LPMO: Lytic polysaccharide monooxygenases
MB: Mossbauer spectroscopy
Moco: Molybdenum cofactor
MPT: Molybdopterin
Mt: *Moorella thermoacetic*
Ma: *Methanococcus aeolicus*
NHE: Normal hydrogen Electrode
PDB: Protein Data Bank
PCET: Proton-coupled electron transfer
Rr: *Rhodospirillum rubrum*
XO: Xanthine oxidase
